# City Magnates and Their Work

**Published:** 1888-11-10

**Authors:** 


// The Hospital

A Weekly Institutional Journal of

Science, Medicine, Nursing, and Philanthropy.

*For Hospitals, Asylums, Medical Practitioners, Students, Nurses, Families, Parishes, Congregations, and the Charitable Public.*

Vol. V. No. 111.] [November 10, 1888.

### City Magnates and their Work.

The new Lord Mayor will next week signalise the commencement of his term of office by taking a public and decided stand on the broad platform of administrative hospital reform. No part of the body politic has a clearer right to be heard on this and other philanthropic questions than the City of London. It is not necessary to claim an ideal perfection for all City institutions and their administration, nor do we take up any such position. But looking at the City and its history, not in the captious spirit of the mole-eyed critic who is always grubbing underground, but with that generous and forbearing temper which distinguishes the truly philosophic student of human affairs, we may affirm without fear that it has deserved almost uniformly well of the country; that at certain great crises its citizens have proved themselves to be made of the genuine stuff out of which English heroes are fashioned, and that it may well hope in the future to emulate and excel all the worthiest traditions of the past.

Sir Sydney Waterlow has undertaken to read a paper at St. Bartholomew's Hospital, under the auspices of The Hospitals Association, on "Sixteen Years of Hospital Sunday: Its Influence upon the Metropolitan Medical Charities." The Lord Mayor will preside on the occasion. No one is better fitted, perhaps no one is so well fitted as Sir Sydney Waterlow to deal with this important and interesting subject. In 1872 Sir Sydney, then Mr. Alderman Waterlow, was Lord Mayor of London. That year was made memorable in the City annals by the institution of the Metropolitan Hospital Sunday Fund and the entertainment, with more than regal splendour, of the Shah of Persia and other Royal and distinguished persons. At the close of his mayoralty Sir Sydney received the honour of a baronetcy. A few years later the treasurership of St. Bartholomew's Hospital became vacant, and he was unanimously appointed by the governors to succeed Mr. Forster White. That honourable appointment he has ever since continued to hold with the greatest advantage to the hospital and its medical school.

It was during the mayoralty of Sir Sydney Waterlow that the late Dr. Wakley, of the *Lancet*, published a series of articles and paragraphs urging the necessity for the formation of a Hospital Sunday Fund in London. The Rev. Canon Miller, who had given such signal aid to the fund in Birmingham, was one of the members of the first-elected council, and rendered important personal service to the Lord Mayor in the original organisation of the movement. It was one of Sir Sydney's conspicuous merits that he insisted upon the thoroughly unsectarian basis of the fund; and that basis has been steadily maintained to the present time. At the close of his year of office as Lord Mayor, Sir Sydney was elected permanent vice-president of the fund. The Lord Mayor for the time being is always president by virtue of his office. The fund therefore is in every respect a City organisation. The fact of his being permanent vice-president has naturally led Sir Sydney to take an unwearying personal interest in each year's collections; and as each succeeding Lord Mayor has delegated to him the supervision of the task of distribution, he has become also a kind of permanent chairman of the distribution committee.

The Hospitals Association, under the auspices of which the meeting will be held, is an organisation which is not sufficiently known to that large portion of the public which charges itself with the support of charitable institutions. Hospitals themselves, in detail and in the mass, are not understood in their actual working as they should be. People who give to charities ought to make some effort to find out how their money is spent. There need be no doubt whatever—we speak from experience—that hospital managers have good intentions. Perhaps it would be impossible to find a single charitable committee in the whole kingdom of whom it could be said with truth that it consisted of unworthy men. But in the disposal of large sums of money, the foolish spending of which involves no evil consequences to the spenders, something more than good intentions is needed. The truth is, that in spite of all the good intentions and self-denying labours of hospital managers, there is in Great Britain a waste of charitable resources which is grievous, because unnecessary. It is of no use blinking the facts or trying to offer plausible explanations. Such blinking and explaining would not mend matters in any way, but would make them worse by adding dishonesty to inefficiency.

The hospital managers who founded The Hospitals Association, and who have hitherto supported it out of their own pockets, established it for no other purpose but to check waste, to develop resources, and to increase efficiency. They knew that in endeavouring to deal with the hospital world, as a whole, they were undertaking a gigantic task. They had a right to expect that their fellow-managers throughout London and the country would rally to their standard. Nor have they been disappointed. It is, perhaps, not too much to say that every hospital manager in

London and the country who brings both intelligence and conscience to his work is in full sympathy or active co-operation with the aims of the Association.

It was not to be expected or desired that an organisation founded to promote such unpopular objects as economy and efficiency would find all men or all women friendly. Some men and some women have been unfriendly. Such things always happen. Real or supposed private and personal interests may have been interfered with, thus giving rise to one kind of opposition. Then there is in certain persons a natural spirit of antagonism to anything and everything of which they themselves have not had either the initiation or the control. Such opposition is not only always to be expected, but is to be welcomed and used. It helps to spread abroad a knowledge of what is being done; and by its accusations of various kinds awakens a spirit of inquiry. Nothing is so favourable to a just and useful movement as publicity and inquiry. The candid spectator, finding a meritorious institution assailed by interested but irresponsible persons, at once concludes that he ought to take his share in stamping out the noxious weeds that grow among the corn, and so takes upon himself the active support of the Association. In this way, to compare secular things with sacred, the blood of the martyrs always becomes the seed of the Church.

It is not necessary to recount in detail the work which has already been accomplished. Among other things may be mentioned the masses of statistics which have been collected and made available, the valuable papers that have been read and published on hospital construction, administration, and finance; and the successful initiation and secure establishment of the National Pension Fund for Nurses. These are examples of what the Association has already carried to a successful issue. It is now engaged in the consideration of still more practical questions in hospital finance and economy as well as in the elaboration of important schemes for the wider diffusion of hospital aid, and for the bringing together of patients and hospitals with a promptitude hitherto unknown. The striking fact about the Association is that it is the only organisation of the kind now in existence. Whilst critics of every degree of competence and incompetence have been recommending this, that, and the other panacea for hospital mismanagement; whilst some have called for a Royal Commission, and others have demanded the charging of hospital expenditure upon the rates, this Association has grappled with urgent problems, one by one, with honesty, capacity, and success.

The meeting on Thursday next, which, as we announce elsewhere, is to be held at St. Bartholomew's Hospital, while it will probably avoid "burning" questions, will be one of great interest and importance. A large gathering of representative hospital managers is expected to be present. The meeting, with the Lord Mayor as president and Sir Sydney Waterlow as reader, may be looked upon as the celebration of the first jubilee of the Hospital Sunday Fund. No one, as we have said, is better fitted to tell the story of the Fund than Sir Sydney Waterlow, and no one is more capable of giving ripe counsel for its future extension and growth. All those who charge themselves with the maintenance of hospitals and all who interest themselves in the noble philanthropic movements of the times should make it their business to take part in the interesting celebration.

